# Improving WASH facilities and practices in Bangladeshi schools: progress and challenges from 2014 to 2018

**DOI:** 10.1080/16549716.2025.2466896

**Published:** 2025-02-19

**Authors:** Farjana Jahan, Noshin Sayiara Shuchi, Afsana Yeamin, Titly Sen, Abul Kasham Shoab, Mahbub-Ul Alam, Supta Sarker, Mehjabin Tishan Mahfuz, Mahadi Hasan, Hasin Jahan, Khairul Islam, Md. Masud Alam, Mahbubur Rahman

**Affiliations:** a Environmental Health and WASH, Health System and Population Studies Division, International Centre for Diarrheal Diseases Research, Bangladesh (icddr,b), Dhaka, Bangladesh; b School of Civil Engineering, University of Leeds, Leeds, UK; c Nutrition Research Division, International Centre for Diarrheal Diseases Research, Bangladesh (icddr,b), Dhaka, Bangladesh; d WaterAid, Dhaka, Bangladesh; e WaterAid, South Asia Regional Office, Dhaka, Bangladesh; f Demography and Health Wing, Bangladesh Bureau of Statistics, Dhaka, Bangladesh; g Global Health and Migration Unit, Department of Women’s and Children’s Health, Uppsala University, Uppsala, Sweden

**Keywords:** School WASH, water, sanitation and hygiene, WASH facilities, environmental hygiene, Bangladesh

## Abstract

**Background:**

In low- and middle-income countries like Bangladesh, inadequate water, sanitation, and hygiene (WASH) practices lead to a higher disease burden among children and hinder their academic performance. However, there have been efforts to improve WASH between 2014 and 2018.

**Objectives:**

The study aimed to investigate changes in WASH facilities and practices in Bangladeshi schools from 2014 to 2018.

**Methods:**

We analyzed pooled data from Bangladesh National Hygiene Survey 2014 and 2018. We performed descriptive analysis, bivariate analysis, and multivariate Generalized Estimating Equation (GEE) to analyze the changes over the four years time period.

**Results:**

Results showed that basic drinking water services increased from 78% in 2014 to 90% in 2018. Schools showed a significant increase in basic sanitation services from 19% in 2014 to 52% in 2018. We discovered that students’ access to water and soap increased from 2014 to 2018, from 21% to 35%. In the GEE model, we found that change in time, non govt urban schools were associated factors with improved basic drinking water services. For basic sanitation services, changes in time, school type and area type were significantly associated higher services. And for basic hygiene services, the associated factors were: schools having hygiene promotion visits, and availability of hygiene brigades at schools managed by students.

**Conclusion:**

WASH services in Bangladeshi schools have improved significantly, yet disparities exist, particularly in government and rural schools. Although students’ knowledge improved, their practices still need improvements through training on proper WASH practices.

## PAPER CONTEXT

- **Main findings:** This study highlights the positive trends in water, sanitation and hygiene situation in Bangladeshi schools, which showed sanitation services in Bangladeshi urban and rural schools improved significantly over the years.
- **Added knowledge:** Moreover, the study found that rural area and government schools still have lower sanitation and hygiene services than urban and non-government schools which demand additional attention.
- **Global health impact for policy and action:** Where current policies and strategies are emphasizing the infrastructural improvement, student’s practices improvements through training on proper hygiene practices should be emphasized more.

## Background

Water, sanitation, and hygiene (WASH) facilities, such as safe drinking water, sanitation, and handwashing facilities, as a component of quality education, can improve students’ attendance in schools [1,2]. Research has shown that school-based WASH interventions can promote a healthier environment for students, which may significantly reduce illness and diarrhea-related absences among school children [3]. Children with lack of proper hygiene and sanitation facilities are more prone to becoming unwell, lowering their learning capacities and interfering with their education [1,4]. However, maintaining proper sanitation and improving hand hygiene in schools continue to be a concern in resource-poor settings, despite increasing efforts [2,5,6]. Lack of basic drinking water services, inadequate WASH practices can contribute to increased disease burden among children, including infectious diseases like diarrhea and pneumonia, which are estimated to kill 1.7 million children annually [7–9]. Students’ WASH practices are strongly influenced by the availability and accessibility of water, sanitation and hygiene facilities, which are not always available to school-aged children in low- and middle-income countries like Bangladesh [1,7,10].

According to SDG indicator 4.a.1, basic WASH services along with other facilities to be provided to all schools and goal 6 includes targets for universal access to safe drinking water (6.1), sanitation and hygiene (6.2) for all [11]. There is still a lack of access to adequate WASH facilities for school going children in Bangladesh [12], the national hygiene survey 2014 showed that only 35% schools had handwashing facilities with soap and water, 84% schools had functional, improved toilet facilities and 80% schools had improved, functional water sources at schools [13]. Literature reveals that despite significant attempts to improve school-going children’s WASH practices, maintaining hygiene may be difficult due to limited water and soap and the deployment of low-cost hand cleaning technologies such as Alcohol-Based Hand Rubs (ABHR) in school settings [14–16]. The unavailability of water, lack of access to hygiene resources inside latrines or handwashing stations, and poor hand hygiene practices may put students at a higher risk of exposure to pathogens [1,13,17]. Proper handwashing helps improving school attendance and reduces preventable diseases [7,9].

Similarly, students are less eager to use toilets that are poorly maintained, and lack adequate and segregated sanitation facilities for both genders [2,18]. Studies have shown that toilet cleanliness has strong correlations with health risks to students and increased school absence, lowering their academic performance [2,19]. Although the Government of Bangladesh committed to achieve 100% safely managed sanitation by 2014, in few cases, schools in Bangladesh either lack the required number of latrines, or the available latrines are locked, with only 50% schools having basic sanitation coverage for students [11,18,20]. Bangladesh standards stipulate a 1:50 ratio of toilet/students, although this isn’t maintained in many of the schools [20,21].

Similarly, the services for drinking water availability are not equal in all schools [21] In schools in Bangladesh, basic service coverage for drinking water was around 83% in 2018 [22]. Access to safe drinking water is critical for children’s health, well-being, and education [23]. However, a report by UNICEF showed that this gap in provision of safe drinking water in Bangladesh affects as many as 8.5 million school children, which can have long term impacts in their cognitive development [23].

To achieve the SDG target, although the Government of Bangladesh adopted short-term and long-term action plans, still, seven percent of schools in Bangladesh don’t have WASH facilities at all [23]. Therefore, there is an urgent need of identifying the gaps in school settings that hinder access to WASH facilities and hygiene behaviors, which are a prerequisite to cut diseases transmitted through water, food, and hand contact [18]. ‘The Water and Sanitation Sector Development Plan (2011–25)’, released by the Government of Bangladesh, highlights the commitment of the Ministry of Primary and Mass Education (MoP&ME) and the Ministry of Education (MoE) to improve hygiene and sanitation in schools [24]. In 2015, the Ministry of Education undertook several strategies to implement the roadmap, which includes the installation of gender-segregated sanitation facilities in co-ed institutions and a collaborative approach towards capacity building of school committees, teachers, and students to monitor WASH services in schools [25]. In 2018, the National Hygiene Survey was again conducted to assess the progress of WASH-related indicators. However, there are few studies that have compared the changes in school WASH services over two different periods of time. Hence, our study aims to evaluate the changes in schools’ WASH facilities and students’ hygiene practices that occurred between 2014 and 2018 using datasets from two nationally representative hygiene surveys.

## Methods

### Data sources

The data for this study was taken from the Bangladesh National Hygiene Baseline Survey 2014 and the National Hygiene Survey 2018, which were conducted by icddr,b and Bangladesh Bureau of Statistics (BBS), subsequently. The surveys were conducted at four levels: household-level hygiene component, school hygiene including MHM, food hygiene in restaurants, and hygiene at hospitals which provided an overview of the handwashing practices and facilities, water, sanitation, and hygiene practices and facilities [3,26]. For this study, we looked into the nationally representative data on the school components that included water, sanitation, and hygiene indicators from urban and rural areas in 2014 and 2018.

### Data sampling procedure

The data for this study was sampled from the National Hygiene Baseline Survey 2014 and the National Hygiene Survey 2018.

#### Sampling for the 2014 dataset

Bangladesh Population and Housing Census 2011 served as the sampling frame and schools were identified using a two-stage cluster sampling procedure. At first, the study team divided Bangladesh into two strata: rural and urban, from where 86,925 rural villages and 10,000 urban mahallas (locality) were selected as representative population clusters. Then using the probability proportional to size (PPS) sampling, a total of 100 clusters were selected considering 50 from rural and 50 from urban areas. One household was selected from each cluster, and the 7 nearest schools were chosen as the study sample, making a total of 700 schools from 100 clusters. The selection was based on the indicator ‘schools having soap and water at handwashing locations’ from a rural school survey [27]. This survey utilized a 10% discrepancy between rural and urban schools, with the design effect of 12.0, power of 0.8, and α of 0.05 to get the desired sample size [13,28]. Based on this estimation, the required sample size was 672 and finally, 700 urban and rural schools were sampled for the hygiene survey.

#### Sampling for 2018 dataset

Using the sampling frame of Bangladesh Population and Housing Census 2011, a total of 176 Enumeration Areas (EAs) were selected from the 293,570 EAs in Bangladesh, utilizing Probability Proportional to Size (PPS) Sampling. The study team organized these EAs into 176 clusters, of which 104 were urban and 72 were rural. Then from each of the 176 clusters, a household was selected to choose 4 nearby schools and generated a sample size of 704 schools (primary and secondary ratio: 2:3). Based on the indicator ‘Hand Washing locations in school with both soap and water available’ from the baseline survey, for both primary and secondary schools, a precision of 6.5 and a design effect of 1.5 was used to get the sample size [26].

### Study site and study population

This section describes the study sites and participant selection criteria for the Bangladesh National Hygiene Baseline Survey 2014 and the National Hygiene Survey 2018.

#### Study site and population in 2014

The schools were eligible if those were government/registered primary or high school and if the headmaster, class teacher gave consent along with the students’ assent. The participants of this study were one headmaster or designated teacher and 4 students from each school enrolled in grades 2 to 10. A total of 700 teachers and 2800 students were interviewed in 2014.

#### Study site and population in 2018

Similar to the survey in 2014, the eligibility criteria were if the schools were primary and secondary schools and if the headmaster, class teacher gave consent. From the eligible schools, interview was conducted with headmasters or designated teachers and four sampled students with equal proportion of boys and girls. For 2018, 704 teachers and 2816 students were finally selected for interview.

### Data collection

The data was collected over two different time periods. Primary data for the Bangladesh National Hygiene Baseline Survey 2014 were collected by a field team of 85 members from icddr,b. Data was collected from March to June 2013, and included face to face survey, spot-check and handwashing demonstrations by school students. Data covered information on availability of drinking water, sanitation, and hygiene practices, and handwashing behavior. The National Hygiene Survey 2018 used in-person interviews, spot checks, and handwashing demonstrations by 25 field teams of trained experts from Bangladesh Bureau of Statistics (BBS) to gather primary data, which took place between March and May 2018 (Table 1).

**Table 1.** Summary of study participants and data collection methods.

Year	Sample size	Participants	Data collection methods
2014	700 schools	• Headmaster or class teacher • 4 students from each school Total: 700 teachers and 2800 students	• Face to face interview • Spot-checks • Handwashing demonstration
2018	704 schools	• Headmaster or teacher • 4 students from each school Total: 704 teachers and 2816 students	• Face to face interview • Spot-checks • Handwashing demonstration

Both surveys used separate questionnaires for school teachers and students. A detailed survey was administered to teachers, focusing on the school facilities’ water sources, sanitation, and hygiene services. For students, a shorter survey was used to collect data on handwashing knowledge and behavior, the availability of school hygiene programs, and the existing water and sanitation facilities. Data collectors also conducted spot checks to evaluate the availability of functional water sources, latrines, and handwashing agents and to observe students’ handwashing practices.

#### Study measures

We grouped the outcomes into three blocks: (i) basic drinking water services (ii) basic sanitation services and (iii) basic hygiene services in schools. We used the WHO/Unicef Joint Monitoring Programme (JMP) definitions for drinking water services, basic sanitation and basic hygiene services [29]. For student’s knowledge and practice regarding basic hygiene services, we identified 5 critical moments for washing hands: Before preparing food, before eating, before feeding a child, after cleaning child’s anus and after defecation [30,31].

For the outcome variables, we considered some factors as independent variables across all three outcomes, which were: time (2018 vs 2014) and area (urban vs rural) [32]. Alongside, for basic drinking water services, we considered School type (govt vs non-govt), Single sex vs combined schools, Number of students, testing for water quality (Arsenic) and surrounding area as independent variables; for basic sanitation services, we considered School type (govt vs non-govt), Schools having improved fixed places for solid waste disposal, appearance of stool around school; and for basic hygiene services we considered hygiene education activities, visit regarding hygiene promotions, and presence of hygiene brigades at school [30,32,33]. Hygiene promotion and hygiene brigade activities were initiated as part of a collaborative capacity-building effort in schools, involving both government and non-government organizations [34]. Interventions like hygiene promotion are initiated in schools by community leaders or non-government organizations, and a student brigade of 24 students from Class Six to Class Nine was formed in each school. In co-education schools, the brigade includes an equal number of boys and girls but in girls-only schools, all 24 members are girls. The brigade is responsible for maintaining latrines and keeping the school premises clean, with help from their teachers [34,35].

### Statistical analyses

We conducted diverse analytical approaches, including descriptive analysis, bivariate analysis, and multivariate Generalized Estimating Equation (GEE) modeling. Given the utilization of the PPS sampling technique for cluster identification, we employed weighted national estimates to account for the rural/urban distribution. To adjust this clustering effect, we multiplied the urban and rural data with the following weight factors: for 2014, Rural clusters-50/86925, Urban clusters-50/10552; and for 2018, Rural clusters-104/86925, Urban clusters-72/10552 [13,26]. We appended the data from 2014 and 2018, subsequently cleaning, documenting and assessing the data in accordance with the 2014 and 2018 survey reports. To explore the changes over the two-time periods, we created a composite ‘time’ variable, where the time point of 2014 was considered as 0 and 2018 was 1.

We conducted a descriptive analysis to assess the characteristics of schools, changes in availability and accessibility of drinking water services, changes in sanitation and hygiene services within schools, changes in environmental hygiene of schools along with students’ knowledge, attitudes, and practices. For continuous variables, we presented the means and standard deviations (SD) and for categorical variables, we presented frequency and percentage. All percentages and means provided are weighted national estimates. We calculated the Proportion Differences (PD) and 95% confidence intervals (CI) for all variables in urban, rural and all schools between 2014 to 2018. We then performed a multivariate GEE model to evaluate the adjusted associations among the independent variables and the outcome variables. We calculated the adjusted coefficient, 95% CI and p-value for the outcome with the exposures of interest. We used the software STATA version 15 to analyze the data.

## Results

**Figure 1.**
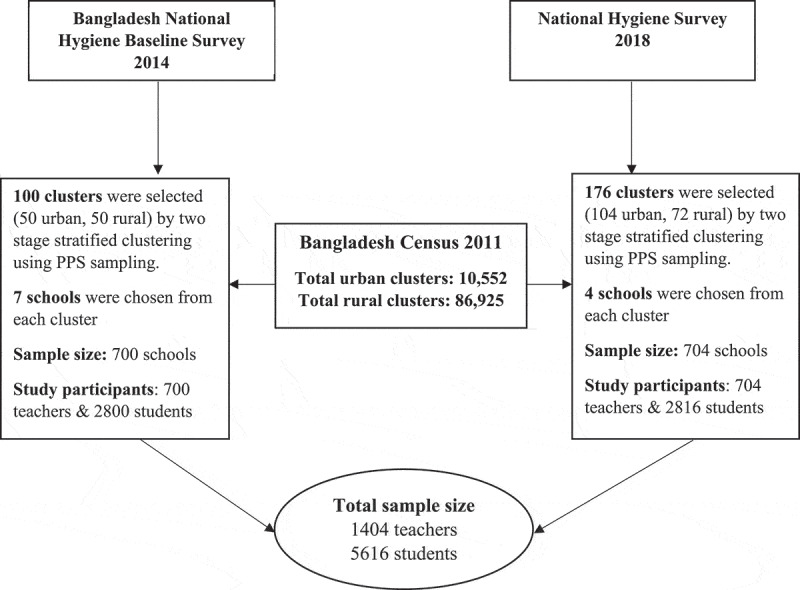
Flow chart showing the selection status of study participants.

The total sample for this study was divided into two groups: teachers and students. The sample size for teachers was 1404 and for students, it was 5616 (Figure 1).

### Background characteristics of schools

The type of schools by education levels were different in 2014 and 2018. The percentage of secondary schools enrolled in the survey increased from 24% to 61% from 2014 to 2018. More non-govt schools were enrolled in 2018 (65%), compared to 33% in 2014. The study participants were divided into two groups-teachers or headmasters and students. The mean number of students interviewed in 2014 survey was 368, which increased to 605 in 2018. The age groups of interviewed students >10 years of age was 62% in 2014, which increased to 94% in 2018 (Table 2).

**Table 2.** Background characteristics of schools.

Indicators		Urban schools n (%*)		Rural schools n (%*)		All schools n (%*)	
		2014 N = 350	2018 N = 288	2014 N = 350	2018 N = 416	2014 N = 700	2018 N = 704
Type of school by education level	Primary	240 (69)	120 (42)	271 (77)	161 (39)	511 (76)	281 (39)
	Secondary	110 (31)	168 (58)	79 (23)	255 (61)	189 (24)	423 (61)
School type	Government schools	229 (65)	124 (43)	237 (68)	183 (44)	466 (67)	307 (45)
	Non-govt schools	121 (35)	164 | (57)	113 (32)	233 (56)	234 (33)	397 (65)
Number of students per school	Primary (Mean, SD)	289.69 (155.06)	330.91 (475.38)	495.89 (434.97)	714.2 (941.98)	309.71 (209.12)	375.21 (562.56)
	Secondary (Mean, SD)	490.5 (317.05)	716.74 (474.25)	963.754 (712.26)	1061.85 (1138.30)	558.99 (431.58)	752.48 (588.30)
Respondent from school	Headmaster	189 (54)	199 (69)	186 (53)	284 (68)	375 (53)	483 (68)
	Teacher	161 (46)	89 (31)	164 (47)	132 (32)	325 (47)	221 (32)
	Student	1,400	1,152	1400	1664	2800	2816
Number of students per school (mean, SD)		642.93 (579.06)	916.99 (1073.01)	335.03 (219.41)	567.41 (510.084)	368 (297.12)	605 (606.62)
		N = 1400	N = 1152	N = 1400	N = 1664	N = 2800	N = 2816
Age group of interviewed students	<10 years	437 (31)	85 (7)	550 (39)	106 (6)	987 (38)	191 (6)
	>10 years	963 (69)	1,067 (93)	850 (61)	1,558 (94)	1,813 (62)	2,625 (94)

*Weighted percentage for rural/urban balance and school size.

### Changes in drinking water source availability, accessibility, and services

**Figure 2.**
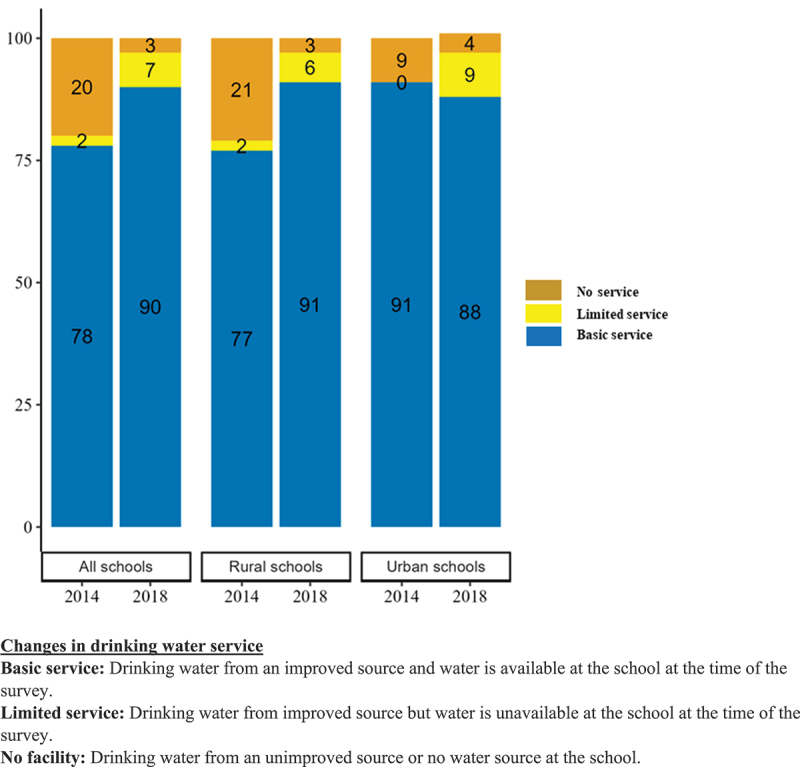
Changes in drinking water services from 2014 to 2018.

The availability of improved drinking water sources has increased significantly from 80% in 2014 to 97% in 2018 (PD: 17, CI 12 to 22); the situation was already better for urban schools (91% to 96%), but there was a significant increase in improved water sources among rural schools from 79% in 2014 to 97% in 2018 (PD: 18; CI 13 to 24). Almost 92% of all schools had water available throughout the year in 2018, this marked a significant upgrade from 79% in 2014 (PD: 12, CI 7 to 18); and the change was more visible in rural schools with a shift from 78% to 92% (Table 3). There was a significant improvement in basic drinking water services in schools, from 78% in 2014 to 90% in 2018 (PD: 12, CI 7 to 18). However, it reduced for urban schools (91% to 88%), but increased for rural schools (77% to 91%). At the same time, number of schools with no service for drinking water has reduced from 20% in 2014 to 3% in 2018 (PD: −17, CI −22 to −12), this reduction was higher for schools in rural areas (Figure 2).

**Table 3.** Changes in drinking water source availability, accessibility, and service s in urban and rural schools of Bangladesh.

Indicators	Urban schools n(%*)		Proportion difference (95% CI)	Rural schools n(%*)		Proportion difference (95% CI)	All schools n(%*)		Proportion difference (%) (95% CI)
	2014 N = 350	2018 N = 288		2014 N = 350	2018 N = 416		2014 N = 700	2018 N = 704	
Availability of improved drinking water sources	318 (91)	277 (96)	5 (1, 10)	275 (79)	403 (97)	18 (13, 24)	593 (80)	680 (97)	17 (12, 22)
Water available in schools throughout the year	318 (91)	260 (90)	−1 (−6, 5)	273 (78)	383 (92)	14 (0.08, 0.2)	591 (79)	643 (92)	12 (7, 18)
Respondents collect drinking water from sources outside school within <20 min distance	141 (40)	11 (4)	−36 (−45, −28)	128 (37)	24 (6)	−31 (−38, −24)	269 (37)	35 (6)	−31 (−38, −25)
Schools tested for water quality (Arsenic)	144 (41)	133 (46)	5 (−8, 19)	201 (57)	234 (56)	−1 (−10, 7)	345 (57)	367 (55)	−1 (−8, 7)
Changes in drinking water services in schools									
Basic	317 (91)	252 (88)	3 (−9, 3)	268 (77)	377(91)	14 (8, 20)	585 (78)	629 (90)	12 (7, 18)
Limited	1 (0)	25 (9)	8 (4, 13)	7 (2)	26 (6)	4 (2, 7)	8 (2)	51 (7)	5 (2, 7)
No facility	32 (9)	11 (4)	−5 (−10, −1)	75 (21)	13 (3)	18 (−24, −13)	107 (20)	24 (3)	−17 (−22, −12)

*Weighted percentage for rural/urban balance and school size.

### Changes in sanitation related service availability, acceptability and student’s perception on latrine use

**Figure 3.**
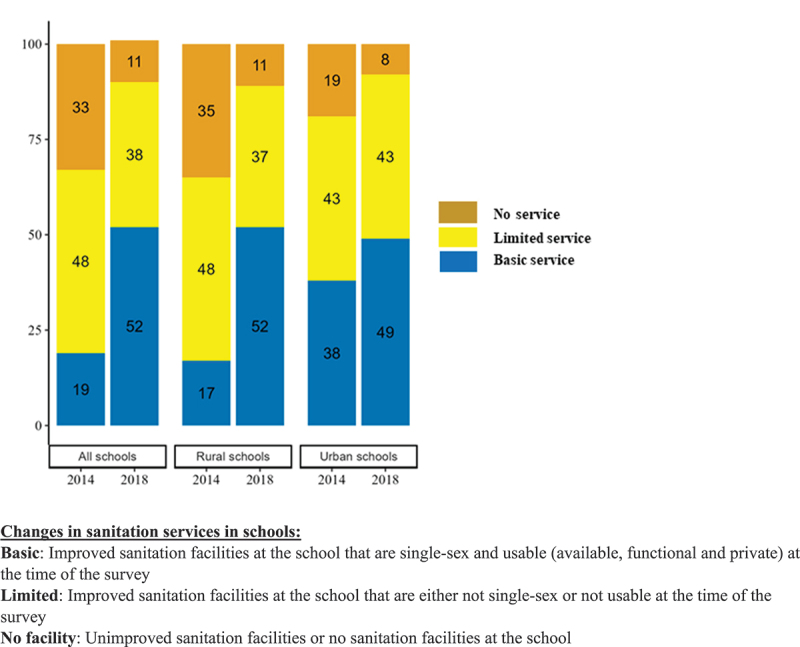
Changes in sanitation services in schools from 2014 to 2018.

The percentage of schools with improved toilets have increased significantly from 90% in 2014 to 99% in 2018 (PD: 9; CI 6 to 13). The basic sanitation services in schools have increased from 19% to 52% in 2014 and 2018 (PD: 33; CI 26 to 39). The increase was much more evident in rural schools where basic sanitation services improved from 17% to 52% (Figure 3). In 2018, almost 74% schools had single sex toilets for female students and teachers, which was a significant improvement from only 22% in 2014 (PD: 52; CI 47 to 58). There was notable increase in schools in rural areas, from 20% to 74% (PD:54, CI 48 to 60), although the rate was still high for urban schools (from 42% in 2014 to 80% in 2018).

At the same time, percentage of schools with improved hand cleansing agent like soaps, detergent increased from 59% in 2014 to 83% in 2018 (PD: 24; CI 17 to 30), the increase was visible in both urban and rural schools. Students’ access to latrines when necessary also increased from 86% to 94% (PD: 8; CI 4 to 12), the findings was similar for both rural and urban schools. In 2014, 62% students perceived that using latrines causes less illnesses, which increased to 66% in 2018 (PD: 27; CI 22 to 32), the knowledge level was similar for schools in rural and in urban areas. These detailed changes are shown in Table 4.

**Table 4.** Changes in sanitation related service availability, accessibility and student’s perception in urban and rural schools of Bangladesh.

Indicators	Urban schools n(%*)		Proportion difference (95% CI)	Rural schools n(%*)		Proportion difference (95% CI)	All schools n(%*)		Proportion difference (%) (95% CI)
	2014 *N* = 350	2018 N = 288		2014 N = 350	2018 N = 416		2014 N = 700	2018 N = 704	
Availability of sanitation services									
Schools with improved latrines	332 (95)	286 (99)	4 (2, 7)	311 (89)	411 (99)	10 (6, 14)	311 (90)	411 (99)	9 (6, 13)
Changes in sanitation service in schools									
Basic service	115 (38)	107 (49)	12 (1, 22)	58 (17)	184 (52)	35 (28, 42)	173 (19)	291 (52)	33 (26, 39)
Limited service	133 (43)	93 (43)	−1 (−12, 10)	165 (48)	132 (37)	−11 (−19, −4)	298 (48)	225 (38)	−10 (−17, −03)
No service	58 (19)	18 (8)	−11 (−17, −4)	118 (35)	39 (11)	−24 (−30, −17)	176 (33)	57 (11)	−22 (−28, −17)
	N = 306	N = 218		N = 341	N = 355		N = 647	N = 573	
Schools with toilets for female students and female teachers (single sex)	127 (42)	174 (80)	38 (30, 47)	67 (20)	261 (74)	54 (48, 60)	194 (22)	435 (74)	52 (47, 58)
School with improved hand cleansing agent (soap, detergent) <30 meter from latrine	246 (70)	256 (89)	19 (11, 26)	203 (58)	342 (82)	24 (0.17, 0.32)	449 (59)	598 (83)	24 (17, 30)
Accessibility to sanitation services									
Teachers responded latrines are accessible throughout the year	343 (98)	287 (100)	2 (0, 3)	327 (93)	412 (99)	6 (3, 9)	670 (94)	699 (99)	5 (2, 8)
Schools with latrine accessible to all students	41 (12)	20 (26)	76 (8, 21)	75 (21)	86 (21)	−1 (−7, 6)	116 (20)	162 (21)	1 (−5, 7)
	N = 1400	N = 1152		N = 1400	N = 1664		N = 2800	N = 2816	
Students having access to latrines when necessary	1233 (88)	1074 (93)	5 (0, 10)	1200 (86)	563 (94)	8 (4, 12)	2433 (86)	2,637(94)	8 (4, 12)
Students’ perception on benefits of latrine use									
Less diarrhea	521 (37)	473 (41)	4 (−4, 11)	461 (33)	588 (35)	2 (−.03, .08)	982 (33)	1061 (36)	3 (−2, 08)
Less illness (without disease specification)	915 (65)	768 (67)	1 (−5, 8)	857 (61)	1103 (66)	5 (0, 10)	1772 (62)	1871 (66)	5 (0, 10)
Less germs	514 (37)	721 (63)	26 (19, 32)	350 (25)	871 (52)	27 (22, 33)	864 (26)	1592 (53)	27 (22, 32)

*Weighted percentage for rural/urban balance and school size.

### Changes in availability, accessibility to hygiene facilities and students’ KAP

#### Availability and accessibility to hand washing facilities

The percentage of schools with handwashing devices within school compound increased from 67% in 2014 to 79% in 2018 (PD: 12; CI 6 to 18), the percentage was still higher for urban schools (88%), compared to rural schools (78%) in 2018. In 2018, 78% of all schools had funds for buying handwashing materials, which increased significantly from 37% (PD: 41; CI 34 to 48) and 79% schools in both rural and urban areas have funds for buying handwashing materials (Table 5).

**Table 5.** Changes in availability, accessibility to hygiene facilities and students’ knowledge, attitude and practice in urban and rural schools of Bangladesh.

Indicators	Urban schools n(%*)		Proportion difference (95% CI)	Rural schools n(%*)		Proportion difference (95% CI)	All schools n(%*)		Proportion difference (%) (95% CI)
	2014 N = 1390	2018 N = 1152		2014 N = 1392	2018 N = 1664		2014 N = 2782	2018 N = 2816	
Availability and accessibility to hand washing facility									
School with handwashing location/devices within school compound	1070 (77)	1,016 (88)	11 (5, 17)	910 (65)	1293 (78)	12 (5, 19)	1980 (67)	2309 (79)	12 (6, 18)
	N = 350	N = 288		N = 350	N = 416		N = 700	N = 704	
Schools with improved handwashing locations with both soap and water	173 (49)	80 (28)	22 (−31, −12)	133 (38)	144 (35)	3 (−11, 4)	306 (39)	224 (34)	−5 (−12, 2)
Schools have fund for buying handwashing materials	176 (51)	228 (79)	29 (21, 37)	124 (35)	324 (79)	42 (35, 50)	300 (37)	552 (78)	41 (34, 48)
Expenditure on handwashing materials (Mean, SD)	137 (135.57)	581 (1930.81)	444 (211.14, 677.74)	103 (108.70)	244 (305.52)	141 (104.09, 178.39)	123 (126.10)	384 (1273.79)	261 (159.22, 362.79)
	N = 1400	N = 1152		N = 1400	N = 1664		N = 2800	N = 2816	
Changes in hygiene services in schools for students									
Basic service	252 (18)	366 (32)	14 (8, 20)	298 (21)	596 (34)	15 (9, 21)	550 (21)	962 (35)	14 (9, 20)
Limited service	775 (55)	605 (52)	−3 (−10, 5)	573 (41)	653 (39)	−2 (−8, 5)	1,348 (43)	1,258 (41)	−2 (−8, 4)
No service	373 (27)	181 (16)	−11 (−17, −5)	529 (38)	415 (25)	−13 (−20, −6)	902 (37)	596 (24)	−13 (−19, −6)
Students’ knowledge, attitude and practice									
Student perceived benefits of washing hands with soap									
Less diseases	1094 (78)	1016 (88)	10 (5, 15)	1063 (76)	1467 (88)	12 (7, 17)	2157 (76)	2483 (88)	12 (8, 16)
Less germs and hands are cleaner	1139 (81)	898 (78)	−3 (−8, 2)	1013 (72)	1255 (75)	3 (−2, 8)	2152 (73)	2153 (76)	2 (−2, 7)
Perceived handwash with soaps is important at 5 critical moments									
Before preparing food	97 (7)	162 (14)	7 (3, 12)	105 (8)	216 (13)	5 (2, 9)	202 (7)	378 (13)	6 (3, 9)
Before eating	1241 (89)	1075 (93)	5 (2, 8)	1194 (85)	1,491 (90)	4 (1, 8)	2435 (86)	2566 (90)	4 (1, 8)
Before feeding a child	47 (3)	73 (6)	3 (0, 6)	27 (2)	74 (4)	3 (1, 4)	74 (2)	147 (5)	3 (1, 4)
After cleaning child’s anus	18 (1)	33 (3)	2 (0, 3)	17 (1)	43 (3)	1 (0, 3)	35 (1)	76 (3)	1 (0, 2)
After defecation	1303 (93)	1046 (91)	−2 (−5, 0)	1308 (93)	1522 (91)	−2 (−4, 0)	2611 (93)	2568 (91)	−2 (−4, 0)
Students wash hands with soaps at 5 critical times									
Before preparing food	36 (3)	113 (10)	7 (4, 11)	37 (3)	101 (6)	3 (1, 5)	73 (3)	214 (6)	4 (2, 6)
Before eating	1177 (84)	1036 (90)	6 (2, 10)	1,116 (80)	1443 (87)	7 (3, 11)	2293 (80)	2479 (87)	7 (3, 11)
Before feeding a child	18 (1)	60 (5)	4 (2, 6)	10 (1)	76 (5)	4 (3, 5)	28 (1)	136 (5)	4 (3, 5)
After cleaning child’s anus	5 (0)	23 (2)	2 (0, 3)	5 (0)	16 (1)	1 (0, 1)	10 (0)	39 (1)	1 (0, 1)
After defecation	1292 (92)	1072 (93)	1 (−1, 3)	1,283 (92)	1566 (94)	2 (0, 5)	2575 (92)	2638 (94)	2 (0, 4)
Students with clean appearance of both hands (spot check)	1,131 (81)	941 (82)	1 (−5, 7)	995 (71)	1234 (74)	3 (−2, 8)	2126 (72)	2175 (75)	3 (−2, 8)
Students washed hands with both water with soap	4 (0.28)	513 (45)	44 (38, 50)	1 (0.07)	881 (53)	53 (47, 58)	5 (0.09)	1394 (52)	52 (47, 57)
Duration of hand washing ≥20 second	267 (19)	581 (50)	31 (24, 39)	260 (19)	847 (51)	32 (26, 38)	527 (19)	1428 (51)	32 (27, 38)

*Weighted percentage for rural/urban balance and school size.

**Figure 4.**
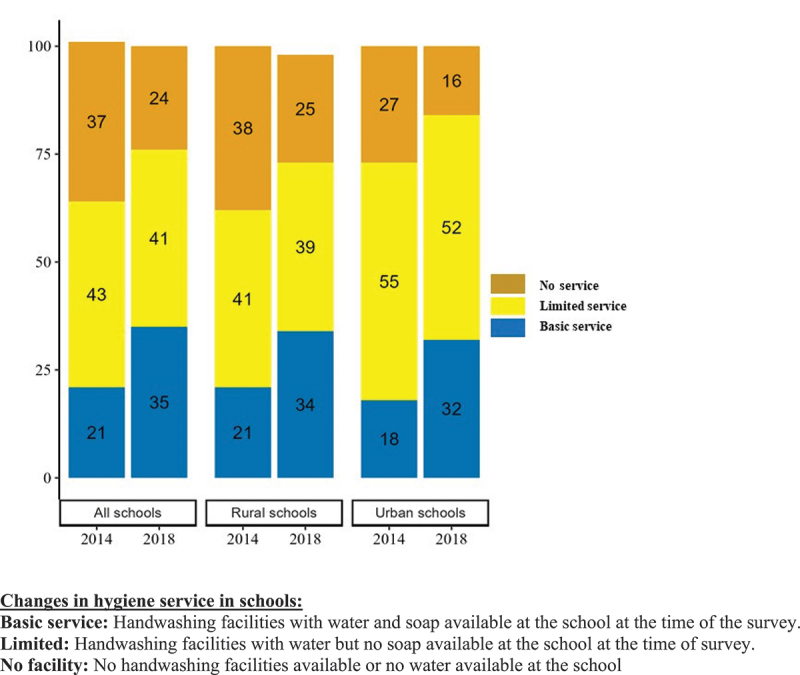
Changes in hygiene services in schools for students between 2014 to 2018.

However, student reported access to basic hygiene services was still quite low for all schools, 21% in 2014 to 35% in 2018 (PD: 14; CI 9 to 20) and the change is similar for both urban and rural schools (Figure 4).

#### Students’ knowledge, attitude and practice on handwashing

88% students in 2018 believed that handwashing with soap causes less diseases, an increase from 76% in 2014 (PD: 12; CI 8 to 16), the change is similar for both urban and rural schools. From the five identified critical moments of handwashing, we compared students’ perception and practices. Students perception on handwashing before eating increased from 86% to 90% (PD:4; CI 1 to 8), and similarly in practice, the percentage increased from 80% to 87% in 2014 to 2018 (PD: 7; CI 3 to 11). This perception and practice were similar for urban and rural schools. In 2018, 91% perceived that handwashing with soap is important after defecation, and 94% students practiced it; the rate of students engaging in handwashing after defecation in rural areas was on par with urban areas. In 2014, overall 19% students practiced handwashing with a duration of ≥20 seconds, which increased to 51% in 2018 (PD: 32; CI 27 to 38), a similar rise in handwashing duration was visible in urban (from 19% in 2014 to 50% in 2018) and rural schools (19% in 2014 to 51% in 2018) (Table 5).

### Changes in environmental hygiene of schools and school hygiene program

In 2018, 65% of all schools were reported to have improved fixed places for disposing solid wastes, which increased from 44% in 2014 (PD: 21; CI 14 to 28), the change was higher for rural schools (PD: 22; CI 14 to 29) than urban schools (PD:17; CI 9 to 26). Though, the overall rate of students using those for dumping solid wastes was still quite low in 2018 (39%), it improved significantly from 2014 (PD:33; CI 27 to 39). This practice was higher among urban school students (53%) in 2018, compared to students in rural schools (38%). Appearance of visible stools inside the school toilet has decreased significantly from 55% to 42% in 2014 to 2018 (PD: −13; CI −19 to −6). In urban schools, it reduced from 64% to 46% (PD: −18; CI −26 to −10) and for rural schools, it reduced from 54% to 42% (PD: −12; CI −19 to −5).

#### Availability of school hygiene program

Books on hygiene were available in 64% schools in 2018, which was an improvement from 21% in 2014 (PD:42; CI 37 to 48). In 2018, a higher percentage of rural schools (64%) had hygiene-related books accessible, in contrast to urban schools (57%). Schools with hygiene education activities also increased from 34% to 43% in the years 2014 to 2018 (PD:8; CI 2 to 15), the improvement was observed in both urban and rural schools. Overall in all schools 35% of students reported that they received hygiene promotion visits in 2018, which was higher than 2014 (23%) (PD: 12; CI 6 to 18) (Table 6).

**Table 6.** Changes in environmental hygiene of schools and school hygiene program.

Indicators	Urban schools n(%*)		Proportion difference (95% CI)	Rural schools n(%*)		Proportion difference (95% CI)	All schools n(%*)		Proportion difference (%) (95% CI)
	2014 N = 350	2018 N = 288		2014 N = 350	2018 N = 416		2014 N = 700	2018 N = 704	
School having improved fixed places for solid waste disposal	193(55)	209(73)	17 (9, 26)	148 (42)	266 (64)	22 (14, 29)	341(44)	475 (65)	21 (14, 28)
Schools with students dumping solid waste properly	42 (12)	152 (53)	41 (33, 49)	21 (6)	157 (38)	32 (25, 38)	63(7)	309 (39)	33 (27, 39)
Schools with classroom and compounds are clean	42 (12)	95 (33)	21 (14, 28)	30 (9)	94 (23)	14 (9, 19)	72 (9)	189 (23)	15 (10, 20)
**Cleanliness of water source and latrine area**									
Water logging in the platform of water source	58 (17)	32 (11)	−5 (−11, 1)	74 (21)	90 (22)	0 (−7, 8)	132(21)	122 (21)	0 (−6, 6)
Area surrounding the water source looked clean	191 (55)	166 (58)	3 (−5, 11)	153 (44)	212 (51)	7 (−1, 15)	344 (45)	378 (52)	7 (−1, 14)
Schools with visible stool inside the functional toilet facility	223 (64)	132 (46)	−18 (−26, −10)	189 (54)	175 (42)	−12 (−19, −5)	412 (55)	307 (42)	−13 (−19, −6)
**Availability of school hygiene program**									
	N = 1400	N = 1152		N = 1400	N = 1664		N = 2800	N = 2816	
Availability of books on hygiene in school	362 (26)	654 (57)	31 (23, 39)	291 (21)	1,073 (64)	44 (38, 50)	653 (21)	1727(64)	42 (37, 48)
Students read books on hygiene from school	251 (18)	592 (51)	33 (26, 4)	185 (13)	946 (57)	44 (38, 50)	436 (14)	1538 (56)	43 (37, 48)
Schools have hygiene education activities	485(35)	451 (39)	5 (−3, 12)	477 (34)	714(43)	9 (10, 16)	962 (34)	1165 (43)	8 (2, 15)
Students received hygiene promotion visits in school	227 (16)	372 (32)	16 (9, 23)	327 (23)	584 (35)	12 (5, 19)	554 (23)	956 (35)	12 (6, 18)
School have hygiene brigade at school	607 (43)	475 (41)	−2 (−11, 7)	667 (48)	766 (46)	−2 (−10, 6)	1274 (47)	1241 (46)	−2 (−9, 5)

*Weighted percentage for rural/urban balance and school size.

### Basic drinking water, sanitation and hygiene services and associated factors

#### Basic drinking water services

The findings of a multivariate GEE model that examined the factors associated to the availability of basic drinking water services showed that changes in time had a positive association with availability of basic drinking water services (coeff: 12; CI: 7 to 18, *p* < .001). The association is still significant when adjusted for school type (Govt vs non-govt, single gender vs co-ed, urban vs rural), number of students, water quality testing and clean area surrounding water source (coeff: 6; CI 1 to 12, *p* < .001). A reduced possibility was associated for basic drinking water services, when the school type was government with a coefficient of −13 (CI: −18 to −8, *p* < .001), the association was significant when adjusted (coeff: −6; CI −11 to −2, *p* < .004). When adjusted, urban schools were more likely to have basic drinking water services compared to rural schools, (coeff: 6; CI: 0.36 to 13, *p* < .05). Schools that were tested for water quality, particularly for arsenic, are more likely to have basic drinking water services, when adjusted (coeff: 27; CI: 22 to 31, *p* < 0.001).

#### Basic sanitation services

The analysis indicates that there has been a significant improvement over time (2014 to 2018) in sanitation services, with a coefficient of 32 (CI: 25 to 39, *p* < 0.001). After adjusting for variables (school type, area, availability of improved fixed places for waste disposal, appearance of visible stool and availability of improved hand cleansing agents), time was still significantly associated (coeff: 29; CI: 22 to 36, *p* < 0.001). Government schools are less likely to have basic sanitation services compared to non-government schools, (coeff: −20; CI: −27 to −12, *p* < 0.001), and when adjusted, the negative association persists, though the magnitude decreases (coeff: −16; CI: −22 to −9, *p* < 0.001).

Urban schools are more likely to have basic sanitation services compared to rural schools, even when considering other variables (coeff: 12; CI: 5 to 19, *p* < 0.001). Schools with an improved fixed place for solid waste disposal are more likely to have basic sanitation services, (coeff: 8; CI: 2 to 13, *p* < .05), but the association is not significant after adjusting. Schools that had improved hand cleansing agents close to latrines were associated with basic sanitation services when adjusted (coeff: 15; CI:8 to 12, *p* < .001).

#### Basic hygiene services for students

After adjusting for other variables, change in time showed a positive association with basic hygiene services in both urban and rural areas (coeff: 14; CI: 9 to 20, *p* < 0.001). When adjusted for other variables: area (urban vs rural), availability of books on hygiene, hygiene education activities, hygiene promotion visits and availability of hygiene brigades, the multivariate analysis indicated an improvement in the availability of basic hygiene services from 2014 to 2018 (coeff: 10; CI: 4 to 15, *p* = 0.003). Urban schools were negatively associated with having basic sanitation at school (coeff: −4; CI: −8 to 1, *p* > .05), but the association was insignificant.

After adjusting, the availability of books on hygiene and hygiene education activities were not significantly associated with basic hygiene services in schools. However, schools that received visits regarding hygiene promotion, showed a positive association with availability of basic hygiene services, when unadjusted (coeff: 17; CI: 13 to 21, *p* < 0.001), and when adjusted, (coeff: 14; CI: 9 to 18, *p* < 0.001). Similarly, schools with hygiene brigades are more likely to have basic hygiene services, and after adjusting, the positive association remains (coeff: 9; CI: 5 to 13, *p* < .001). The analysis is detailed in Table 7. These hygiene brigades are targeted to help the students lead and manage hygiene related issues by building their capacity.

**Table 7.** Basic drinking water, sanitation and hygiene services and associated factors.

Outcome variable	Independent variable	Unadjusted value (95% CI)	p-value	Adjusted model specification	
				Coefficient (β) (95% CI)	p-value
**Basic drinking Water facilities (model1)**					
Basic drinking water service in schools	Time (2018 vs 2014)	12 (7, 18)	**<0.001**	6 (1, 12)	<0.001^*^
	School type (Govt vs non-govt)	−13 (−18, −8)	**<0.001**	−6 (−11, −2)	0.004^*^
	Single gender vs co-educational schools	16 (10, 21)	**<0.001**	5 (−0.10, 11)	0.054
	Area (Urban vs rural)	7 (2, 12)	**0.003**	6 (0.36, 13)	0.038^*^
	Number of students	0.02 (−.002, .05)	0.075	0.01 (−0.002, 0.02)	0.092
	Schools tested for water quality (Arsenic)	30 (24, 36)	**<0.001**	27 (22, 31)	<0.001^*^
	Area surrounding the water source looked clean	21 (15, 27)	**<0.001**	14 (10, 18)	<0.001^*^
**Basic sanitation services (model2)**					
Basic sanitation services in schools	Time (2018 vs 2014)	32 (25, 39)	**<0.001**	29 (22, 36)	<0.001^*^
	School type (Govt vs non-govt)	−20 (−27, −12)	**<0.001**	−16 (−22, −9)	<0.001^*^
	Area (Urban/rural)	11 (4, 18)	**<0.001**	12 (5, 19)	<0.001^*^
	Schools having improved fixed places for solid waste disposal	8 (2, 13)	0.005	3 (−2, 9)	0.206
	Appearance of visible stool inside the latrine	3 (−3, 8)	0.333	5 (−0.90, 10)	0.102
	School with improved hand cleansing agent (soap, detergent) <30 meter from latrine	20 (13, 26)	**<0.001**	15 (8, 22)	<0.001^*^
**Basic hygiene services-for student (model3)**					
Basic hygiene services for students at school	Time (2018 vs 2014)	14 (9, 20)	**<0.001**	10 (4, 15)	<0.001^*^
	Area (Urban/rural)	−4 (−8, 1)	**0.118**	−2 (−6, 1)	0.204
	Books on hygiene services at schools	11 (7, 15)	**<0.001**	1 (−3, 6)	0.591
	Schools having hygiene education activities	12 (8, 17)	**<0.001**	5 (−0.46, 10)	0.074
	Visit regarding hygiene promotion in school	17 (13, 21)	**<0.001**	14 (9, 18)	<0.001^*^
	School having hygiene brigade at school	12 (8, 16)	**<0.001**	9 (5, 13)	<0.001^*^

*(Statistically significant ≤ 0.05).

## Discussion

The aim of this study was to report the changes in basic drinking water, sanitation, and hygiene services in schools from 2014 to 2018, analyzing improvements and challenges at the national, rural, and urban levels. Bangladesh has achieved significant progress in ensuring availability of basic WASH services improved significantly in all schools from 2014 to 2018; with drinking water services increasing from 78% to 90%, sanitation services from 19% to 52%, and hygiene services for students increasing from 21% to 35%. Access to these facilities in schools can help achieve the SDG Goal no 4, which aims to ensure inclusive and quality education for all and promote lifelong learning [11]. Although our analysis revealed an improvement in the overall scenario, there are still considerable gaps in the availability of basic services between urban and rural areas.

According to the global estimates, 71% schools had basic drinking water services [11], and our findings showed that in Bangladesh, the rate was substantially higher at 90%. Similar to our findings, another study conducted in Bangladesh showed a 95% availability of basic drinking water [21], while adding that services provided at basic levels doesn’t take into account the safety of the water supplied [21,36]. As presented in our study, 91% rural schools had basic drinking water, compared to urban schools (88%) in 2018. The change was significant in rural areas due to an increased installation of tube wells and hand pumps, as well as majority of the aid in 2017–18 went to basic drinking water access, mostly for rural areas [37,38]. Similar school-based studies in in Ethiopia and Pakistan showed almost 75% and 58% schools had basic drinking water service [39,40]. However, this information on selective indicators lack comprehensive data on all three areas of WASH in school settings, which makes it difficult to compare the findings.

The significant improvements in Bangladesh can be explained by rigorous initiatives taken by government of Bangladesh, such as installing tube wells, hand pumps, arsenic testing etc. over the years, but due to lack of enough resources and poorly designed infrastructures, maintenance has always been an issue [41–44]. Our study indicated a substantial improvement in basic sanitation services, 52% in 2018, still below the global estimate of 72% [11].

Although a significant percentage of schools might fall behind, our findings indicated a relatively better condition compared to countries with similar income levels, such as Pakistan (19.3%), and Nigeria (38%) [11]. In 2015, the Ministry of Education issued a national circular to improve WASH conditions in secondary and higher secondary schools [20], but with no separate budget allocation [12]. This disparity was reflected in a significant percentage of schools lacking basic sanitation facilities. In contrast, schools in some other low-income countries such as Togo (79%) and Ethiopia (61.3%) had better sanitation facilities [11,39], highlighting the effectiveness of proper fund allocation, community engagement in development and monitoring WASH services, and school-based WASH clubs [39]. Along with availability, accessibility is a major concern; which improved for students from 86% to 94% for both urban and rural schools. We found that living area of students was a significant factor in determining basic sanitation facilities, with rural schools having better services than urban schools. This finding contrasts with a previous Indian study, where rural areas were lagging far behind in basic sanitation facilities [45]. Furthermore, our findings indicated that government schools were less likely to have basic sanitation services, despite the national circular. This suggests the need of establishing maintenance services for sanitation, even after funding has been provided [2].

In 2018, the basic hygiene services stood at 35%, after an improvement from 21%. This finding aligns with a report from UNICEF in the same year, and it was comparable to hygiene facilities in countries with similar income levels like Lebanon, Cambodia and Ethiopia [39,46]. Along with service availability, handwashing practice with soap and water among students also improved from <1% to 52% in 2014 to 2018. This finding is consistent with a similar school based study in Nigeria [47]. Further analysis revealed several potential sub-determinants that might have contributed to this shift in behavior, including knowledge about the benefits of handwashing practice, the availability of cost-effective handwashing agents such as soap or ABHR at latrines or handwashing stations, and awareness of the risks of poor handwashing practices [10,16].

However, despite improvements, these hygiene services were lower in urban schools compared to rural schools, consistent with the findings of JMP [48]. Some of the barriers to proper handwashing facilities and practices, may include lack of soap and water, forgetfulness of students, and students being in a public institution [8,9,47]. Provision of hygiene related education was found to be associated with good handwashing practice among students, as shown in other studies in Bangladesh as well [30,49]. While it is essential to ensure hygiene education and adequate access to water and soap to enhance hygiene practice, a study conducted in Uganda emphasized access to low-cost hand hygiene resources, such as ABHR, to improve hand hygiene behavior, particularly in resource-constrained areas where water and soap are lacking [50]. Nearly 94% demonstrated handwashing practices after defecation, reflecting other findings in Bangladesh [30], and showing improved situation compared to China, Ghana, and Cameroon [7–9].

Poor WASH services may pose significant risk of spread of several infectious diseases [39]. Improving basic WASH services in schools can help students develop good hygiene and sanitation practices, as shown in previous studies done in Bangladeshi and Kenyan schools [5,51,52]. Several political, social and economic differences which has influenced the basic WASH services in different schools of Bangladesh [32]. This further demonstrates the need of proper WASH facilities across all schools in urban and rural areas, coupled with proper maintenance and management.

Since access to standard school WASH (Water, Sanitation, and Hygiene) services is interlinked with students’ academic performance and their physical and mental development, a comparison of findings from the National Hygiene Surveys of 2014 and 2018 may serve as a critical resource for policymakers, government and non-government stakeholders, and school teachers. The evidence generated from this study will guide policymakers in identifying service gaps in urban and rural schools and help them plan meticulously to deliver inclusive WASH services to students from diverse academic backgrounds; thereby maximizing implementation impact [33]. Additionally, school hygiene management committees and teachers responsible for monitoring school WASH facilities and managing funds for hygiene service provision will be able to mobilize resources more effectively based on the study’s findings [33,53]. This review will also inform the government and non-government WASH stakeholders on strengthening WASH monitoring and capacity-building initiatives to be taken in schools from different geographical locations of Bangladesh [53]Furthermore, this document will advocate for creating a safer and healthier environment, particularly in educational settings, to ensure students’ well-being and attain SDG goals.

## Strength and limitations

This study presents evidence-based conclusions by evaluating changes in drinking water, sanitation, and hygiene services in Bangladeshi schools- both urban and rural between 2014 and 2018. The changes are shown throughout two time periods and among a large sample size, which enhances the data’s credibility. The insights gained from the study can serve as valuable support for the development of more effective policies. These findings can help policymakers make informed decisions regarding designing, implementing, and improving context specific initiatives related to WASH facilities in schools.

The absence of data on parental education, work position, family income levels, and so on in the datasets was a limitation, as these might have potentially provided more insight into the behaviors of the pupils. Furthermore, it is challenging to determine causal correlations because the study relies on cross-sectional data.

## Conclusion

The basic WASH services in schools in Bangladesh have improved significantly over the years, with a noticeable improvement in basic drinking water, sanitation and hygiene services in both rural and urban schools. However, basic drinking water and sanitation services were still comparatively lower for government schools, and schools located in rural areas. Students’ knowledge on benefits of latrine use and handwashing practices still needs much improvement. WASH facilities and hygiene services can have significant impact on the wellbeing and academic achievements of school going children. Trainings on behavioral interventions need to be provided while ensuring education on proper WASH practices, to promote behavior change among students. Providing these services may benefit students’ behavior, promoting their health and academic success. An integrated approach should be adopted, while making targeted investments on school WASH services, especially in rural areas. Along with policy implementation, regular monitoring is required to achieve the SDG goals 6 and 4. This can be achieved through collaborative approaches including government agencies, NGOs, and private organizations with the necessary resources and expertise.
